# Role of Fascin-1 in cervical cancer metastasis via Wnt/β-catenin pathway activation

**DOI:** 10.17305/bb.2025.12114

**Published:** 2025-03-14

**Authors:** Yan Wang, Wei Cui, Dong-Mei Chu

**Affiliations:** 1 Department of Gynecology, The Fourth Hospital of Shijiazhuang, Shijiazhuang, Hebei Province, China

**Keywords:** Fascin-1, cervical cancer, CC, activating Wnt/β-catenin pathway

## Abstract

This investigation delves into the impact of Fascin-1, a protein known for its role in actin bundling and its association with metastatic enhancement, on the advancement of cervical cancer (CC). Elevated levels of Fascin-1 have been observed in metastatic carcinomas, but its impact on gene regulation in CC has not been thoroughly studied. Our research demonstrates a marked elevation in the expression of Fascin-1 within tissues affected by CC. Experiments employing both overexpression and knockdown methods revealed that Fascin-1 plays a critical role in promoting the proliferation and mobility of CC cells *in vitro*. Correspondingly, reducing Fascin-1 levels led to a marked decrease in tumor growth and metastatic spread *in vivo*. At the molecular level, diminishing Fascin-1 expression resulted in decreased β-catenin and C-myc RNA and protein levels. This implies that Fascin-1 could intensify the progression of CC by influencing the Wnt/β-catenin signaling cascade. This study not only elucidates the mechanism by which Fascin-1 contributes to the advancement of CC but also proposes a novel approach for therapeutic intervention.

## Introduction

Cervical cancer (CC) is one of the most prevalent malignancies among women and ranks as the fourth most common cancer worldwide [1]. Epidemiological studies consistently show that most CC cases are caused by high-risk strains of human papillomavirus (HPV), particularly HPV16 and HPV18 [2]. However, the incidence of CC in women under 65 has risen due to the increasing prevalence of HPV and inadequate screening measures [3]. The primary treatment modalities for CC remain surgery, radiation therapy, and chemotherapy [4–6]. However, for patients with metastatic disease, treatment options are limited due to the heterogeneity of clinical manifestations [7]. Therefore, identifying more effective therapeutic targets and prognostic markers for CC is of paramount importance.

In the vast majority of metastatic carcinoma types, Fascin (Fascin-1), a protein that bundles actin in a monomeric form, is significantly elevated [8, 9]. Recent oncology research has shown increasing interest in Fascin-1, particularly due to its emerging role in the progression of several aggressive metastatic cancers. Notably, its involvement in various malignancies has been well established. For example, Fascin-1 regulates the direct binding of YAP1/TEAD to PFKFB3, promoting its transcription and thereby enhancing glycolysis and the progression of lung cancer [10]. In ovarian cancer, FSCN1 promotes epithelial–mesenchymal transition by increasing Snail1 [11]. The centrality of Fascin-1 in the molecular pathways of these cancers underscores its potential as both a biomarker and a therapeutic target. Ongoing research continues to explore its role in improving diagnostic precision and treatment efficacy for these challenging malignancies [12–15]. In addition to its well-documented role in metastatic spread, Fascin-1 also contributes to increased chemoresistance and the stemness of cancer cells [16, 17]. Studies suggest that Fascin-1 expression may be influenced by other molecular factors. For instance, miRNA-145-5p is downregulated in CC tissues and shows a significant correlation with tumor grade and lymph node metastasis. As a potential tumor suppressor, miRNA-145-5p may regulate Fascin-1 expression, thereby influencing CC invasion and metastasis [18]. Furthermore, immunohistochemical (IHC) analysis has shown that Fascin-1 expression is relatively low in normal cervical squamous epithelial tissue. However, its positive rate increases significantly in low-grade intraepithelial neoplasia (CIN1), high-grade intraepithelial neoplasia (CIN2-3), and CC tissues, with no significant differences among these three conditions [19]. This pattern suggests that Fascin-1 plays a complex role in cancer initiation and progression. However, the precise mechanisms underlying its function in CC require further investigation.

## Materials and methods

### General

All methods were carried out in accordance with ARRIVE guidelines and regulations.

### Cells and reagents

The Hela and C33A human cervical cell lines were purchased from Procell Life Science & Technology Co., Ltd. (Wuhan, China) and cultured in RPMI 1640 and MEM media, respectively, both supplied by Meilunbio (Dalian, China). The human embryonic kidney cell line HEK293T, which expresses the SV40 large T-antigen, was grown in MEM from Meilunbio. All media were supplemented with 10% FBS (Biological Industries, Israel), and cells were incubated at 37 ^∘^C in a humidified environment with 5% CO_2_. Antibodies used in this study were sourced from various suppliers: anti-FLAG (M2, F3165) and anti-β-actin (A1978) from Merck KGaA (Darmstadt, Germany), while anti-Fascin-1 (ab126776), anti-β-catenin (ab32572), anti-c-Myc (ab32072), and anti-Ki67 (ab15580) were obtained from Abcam Inc.

### RNA interference

To generate Fascin-1 knockdown cell lines, short hairpin RNA (shRNA) plasmids were used in lentivirus-mediated interference. Complementary sense and antisense oligonucleotides encoding shRNAs targeting these two genes were synthesized, annealed, and cloned into the pLKO.1 vector (with the empty pLKO.1 vector serving as the control). Additionally, a FLAG-Fascin-1 expression plasmid was cloned into the pCDH-CMV-MCS-EF1-copGFP vector to overexpress Fascin-1, with the empty pCDH-CMV-MCS-EF1-copGFP vector serving as the control.

The associated sequences were as follows:

sh*FSCN1*-1: 5′- GCGAGTCTGGCACCTCTTT-3′

sh*FSCN1-*2: 5′- GAGCCTTATTTCTCTGGAA -3′

Lentiviral vectors were co-transfected into HEK293T cells along with packaging vectors—PAX2 and pMD2.G for shRNA, and PAX8 and VAVG for FLAG-FSCN1—using PEI (Polysciences, 23966, USA). Infectious lentiviral particles were harvested 24 and 48 h post-transfection, filtered through 0.45 µm PVDF filters, and transduced into target cells.

### Lentiviral infection

During the passage of HeLa and C33A cells, the medium, virus solution, and Polybrene (final concentration: 10 µg/mL) were added to the cells and mixed uniformly. The cells were then incubated in a cell culture incubator for 48 h. After incubation, the medium was replaced with fresh medium containing puromycin (1 µg/mL) to eliminate non-infected cells. Meanwhile, a separate dish of uninfected cells was treated with the same concentration of puromycin as a control. When all cells in the control group were completely eliminated, while most lentivirus-infected cells survived, the surviving cells were considered a stable cell line expressing the target gene. Subsequently, the puromycin concentration in the medium was halved for continued culture.

### RT-qPCR

Quantitative PCR (qPCR) was used to measure gene expression in HeLa and C33A cells. RNA was extracted using Trizol (Yeasen, China), converted to cDNA with the Hifair^®^ III Kit (Yeasen, China), and analyzed using a LightCycler^®^ 480 system with SYBR Green PCR Mix (Bimake, USA). Gene expression levels were normalized to GAPDH and calculated using the 2^-ΔΔCt^ method.

Primers included: for Fascin-1, forward (F) GACACCAAAAAGTGTGCCTTCCG, reverse (R) CAAACTTGCCATTGGACGCCCT; for β-Catenin, F: CACAAGCAGAGTGCTGAAGGTG, R: GATTCCTGAGAGTCCAAAGACAG; and for c-Myc, F: CCTGGTGCTCCATGAGGAGAC, R: CAGACTCTGACCTTTTGCCAGG.

### Western blotting

Proteins were equally loaded and subjected to 10% SDS-PAGE, followed by transfer to PVDF membranes. The membranes were incubated overnight at 4 ^∘^C with primary antibodies, then with secondary antibodies (1:5000, KPL) for 2 h at room temperature. Protein bands were detected using a luminol-based Western blot assay (Santa Cruz Biotechnology, USA).

### MTT assay

Cell viability was assessed using the MTT assay, which utilizes 3-(4,5-dimethylthiazol-2-yl)-2,5-diphenyl-2H-tetrazolium bromide. In this method, 2000 cells were suspended in 100 µL of either MEM or RPMI 1640 supplemented with 10% FBS and plated in a 96-well plate. The cells were then incubated for 24, 48, 72, or 96 h. Following treatment with 0.5 mg/mL MTT in serum-free MEM or RPMI 1640, the plates were incubated at 37 ^∘^C for 4 h. After incubation, the medium was removed, and the cells were lysed with 110 µL of DMSO for 30 min at room temperature. Absorbance was then measured at 490 nm.

### Colony formation assay

In this study, a long-term colony formation assay was performed. Each well of a six-well plate was initially seeded with 500 cells, and the medium was refreshed every three days to maintain optimal growth conditions. After two weeks of growth, the cells were rinsed twice with phosphate-buffered saline (PBS) and then fixed with 4% paraformaldehyde for 15 min. Colonies were subsequently stained with 0.1% crystal violet solution (Solarbio, Beijing) for another 15 min. Finally, the stained colonies were photographed for analysis.

### Migration and invasion assays

In this experiment, a cellular suspension of 5 × 10^4^ cells was prepared in either MEM or RPMI 1640 medium, both without FBS. This suspension was then carefully added to the upper chamber of a 24-well plate, where each well had been pre-coated with a 50 µL layer of Matrigel (BD Biosciences, Franklin Lakes, NJ, USA). (No Matrigel was used in the migration experiment.) The lower wells were filled with 500 µL of MEM or RPMI 1640 medium supplemented with 10% FBS. After a 24-hour incubation, cells that had migrated to the lower surface of the membrane were fixed, stained with 0.1% crystal violet for visualization, and quantified under a microscope.

**Figure 1.**
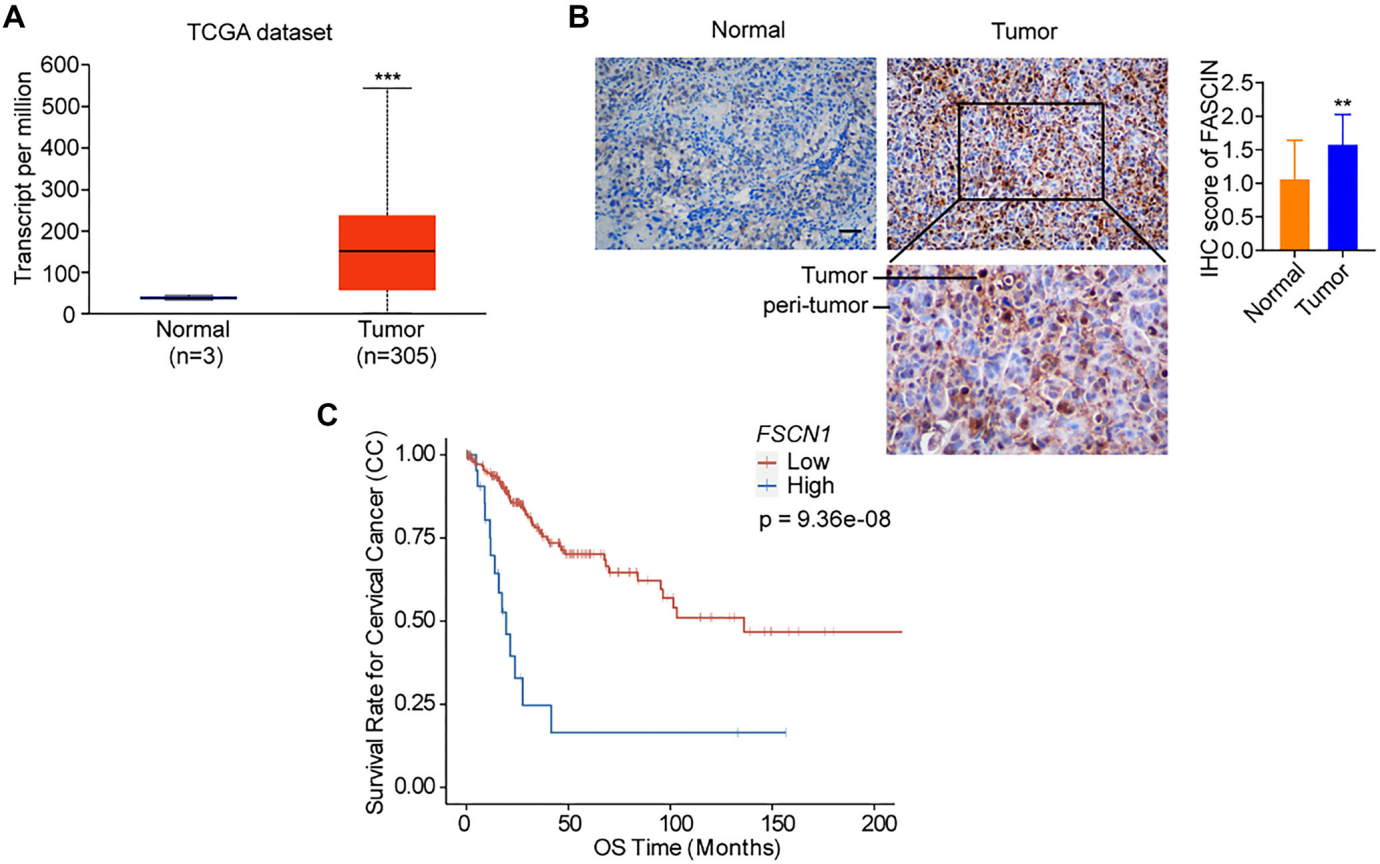
**Elevated expression of Fascin-1 in cervical cancer.** (A) Comparison of *FSCN1* mRNA levels in cervical cancer vs normal tissues based on TCGA data analysis. (B) Fascin-1 immunohistochemical staining in a tissue microarray encompassing cervical cancer and adjacent non-carcinoma tissues. Scale bar represents 100 µm. Statistical analysis of Fascin-1 expression (IHC-score) was conducted on 30 samples from cervical cancer tissues and 25 samples from adjacent non-carcinoma tissues. Significance denoted as ***P < 0.001 (Student’s *t*-test). (C) Kaplan–Meier analysis of the overall survival rate of cervical cancer patients with high or low expression of Fascin-1 (https://smuonco.shinyapps.io/PanCanSurvPlot/). IHC: Immunohistochemical.

### Mouse xenograft models

In this experiment, batches of five million HeLa cells were genetically modified with either Fascin-1-targeting shRNAs or non-targeting control shRNAs. These modified cells were then subcutaneously injected into groups of female athymic nude mice (BALB/c strain) obtained from Charles River Laboratories. Each group consisted of six mice, aged 6–8 weeks. For the cisplatin administration experiments, mice received intraperitoneal injections of cisplatin at a dosage of 7.5 mg/kg, administered twice weekly. Tumor growth was monitored every three days using vernier calipers, and tumor volumes were calculated using the formula: *V* ═ 1/2 × length × width^2^. After four weeks, the mice were humanely euthanized, and the tumors were excised and documented through photography. In a separate metastasis study, another set of mice received intravenous injections of two million HeLa cells, suspended in 100 µL of PBS, directly into their tail veins. These mice were also euthanizedafter four weeks, and their lungs were examined for metastases. All animal experimental protocols were approved by The Fourth Hospital of Shijiazhuang (No. SJZDSYY2023-359).

### Tissue embedding and frozen section

The tumor was separated and washed twice with PBS buffer. After removing excess surface moisture, the tissue was cut into 1 cm^3^ pieces and placed in a tin foil box. The optimal cutting temperature (OCT) embedding agent was then added evenly and slowly around the tissue until it was fully submerged. The embedded tumor tissue was first placed at −20 ^∘^C to freeze into a solid state before being transferred to −80 ^∘^C for long-term storage. Before sectioning, the cryostat was pre-cooled, and the frozen tissue was quickly transferred to the sectioning machine. The blade was secured, and the specimen holder was coated with OCT before placing the sample in a position parallel to the blade. The tissue was sectioned at a thickness of 7 µm and flattened onto slides. After air-drying at room temperature, the slides were transferred to −80 ^∘^C for long-term storage.

### Dual-Luciferase Reporter gene assay

To assess Wnt pathway activation, the indicated plasmids were transfected into CC cells along with the TCF signaling reporter pTop-Flash and its negative control, pFop-Flash. Additionally, cells were co-transfected with the internal control plasmid pSV40-Renilla. After 48 h, luciferase activity was measured using the Dual-Luciferase Reporter Assay System (Promega Corporation, Madison, WI, USA).

**Figure 2.**
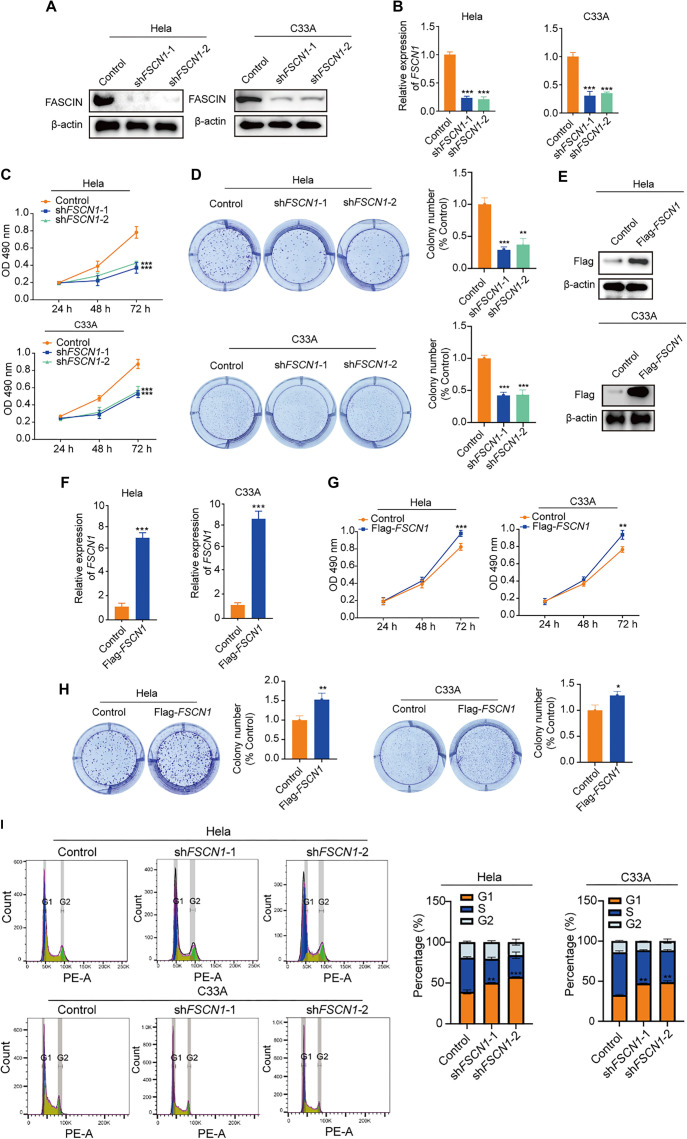
**Impact of Fascin-1 on *in vitro* growth of cervical cancer cells.** (A) Assessment of Fascin-1 levels in Hela and C33A cells through Western blot, post-infection with lentiviruses carrying either *FSCN1*-targeting shRNAs or control shRNAs; (B) RT-PCR analysis of *FSCN1* mRNA levels when knockdown FASCIN; (C) Evaluation of cell proliferation in Hela and C33A cells with *FSCN1* or control shRNA expression, using MTT assays; (D) Growth of Hela and C33A cells expressing respective shRNAs, cultured for 10 days and stained with crystal violet; colony counts were recorded; (E) Western blot analysis of cell lysates from Hela and C33A cells stably expressing FLAG-*FSCN1* empty vector; (F) RT-PCR analysis of *FSCN1* mRNA levels when overexpressed *FSCN1*; (G) Proliferation analysis via MTT assays in cells with either control or *FSCN1* overexpression; (H) Analysis of colony formation in cells with indicated treatments; (I) Cell cycle experiments in indicated cells. Data for Figure 2B–2D and 2F–2I are presented as mean ± SD, *n* ═ 3; ***P < 0.001, **P < 0.01, *P < 0.05 (Student’s *t*-test). shRNA: Short hairpin RNA.

### IHC analysis

The UltraSensitive SP kit (MXB) was used for IHC staining. Tissue sections were fixed, blocked, and incubated with the corresponding primary antibody (Proteintech, 66321-1-Ig, 1:50), followed by a secondary antibody and DAB staining. The slides were then counterstained with hematoxylin and mounted. The IHC score was determined based on staining intensity and the percentage of stained cells. Staining intensity was scored as follows: 0 ═ no detectable staining, 1 ═ weak staining, 2 ═ moderate staining, and 3 ═ strong staining. The percentage of positive cells ranged from 0% to 100%. The final IHC score was calculated by multiplying the intensity score by the percentage score, yielding a total range of 0–3. Tissue sections were independently examined and scored by two investigators who were blinded to the clinicopathological data.

### Ethical statement

All animal experimental protocols were approved by The Fourth Hospital of Shijiazhuang (No. SJZDSYY2023-359).

### Statistical analysis

The analysis was performed using a two-tailed paired Student *t*-test to compare control and treated samples. The data were presented as the means ± standard deviation (SD) from at least three independent assays. Differences were considered significant when the *P* value was less than 0.05 (*P < 0.05, **P < 0.01, ***P < 0,001). Statistical analyses were conducted using SPSS 18.0 and GraphPad Prism 10.3.0.

## Results

### Fascin-1 is upregulated in CC tissues

To elucidate Fascin-1’s role in the progression of cervical carcinoma, we analyzed its expression using RNA-seq data from the TCGA database. Our findings revealed a significant increase in Fascin-1 mRNA levels in CC tissues compared to normal cervical cells, as shown in Figure 1A. Additionally, IHC staining was performed on 30 CC tissue samples and 25 non-cancerous tissue samples to assess Fascin-1 protein expression. This analysis revealed a markedly higher Fascin-1 protein presence in cancerous tissues compared to normal tissues, as illustrated in Figure 1B. Furthermore, Kaplan–Meier survival analysis based on TCGA-CESC data indicated that CC patients with high Fascin-1 expression had poorer overall survival rates. These results suggest that Fascin-1 is highly expressed in CC and may play a role in disease progression.

**Figure 3.**
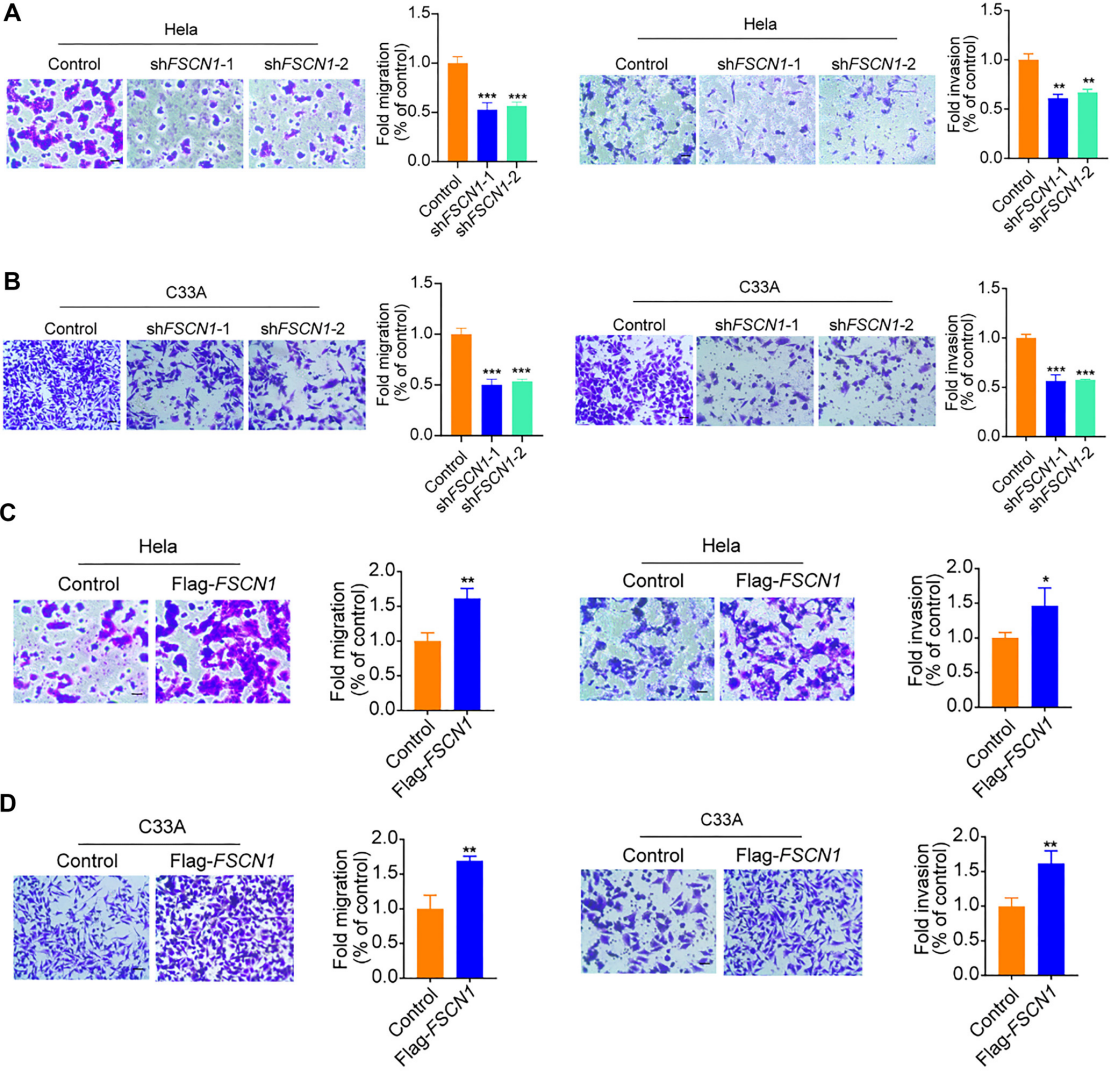
**Enhancement of cervical cell migration by Fascin-1 *in vitro*.** (A and B) Migration and invasion capacities of Hela and C33A cells, with either *FSCN1* shRNAs or control shRNAs, were assessed through respective assays; (C and D) Cells overexpressing either empty vectors or FLAG-tagged *FSCN1* were analyzed for migration and invasion abilities. Scale bars represent 100 µm. Statistical data are presented as mean ± SD (*n* ═ 3); ***P < 0.001, **P < 0.01, *P < 0.05 (Student’s *t*-test).

### Fascin-1 promotes the growth of CC cells *in vitro*

Our research aimed to elucidate the role of FSCN1 in CC progression. To achieve this, we developed lentiviral vectors carrying either control shRNAs or shRNAs targeting FSCN1. Hela and C33A cells were infected with these lentiviruses, resulting in stable expression of the respective shRNAs. The successful knockdown of FSCN1 was confirmed via qRT-PCR and Western blot analysis (Figure 2A and 2B). Using these cell models, we assessed the functional impact of FSCN1 depletion. MTT assays revealed that FSCN1 knockdown significantly reduced the viability of both Hela and C33A cells compared to controls (Figure 2C). Additionally, colony formation assays further confirmed the inhibitory effect of FSCN1 depletion on cell growth (Figure 2D). Conversely, overexpression of FSCN1 in CC cells (Figure 2E and 2F) led to enhanced cell proliferation, as demonstrated by MTT and colony formation assays (Figure 2G and 2H). Furthermore, cell cycle analysis indicated that FSCN1 knockdown induced G1-phase arrest in CC cells. Taken together, these findings highlight a crucial role of FSCN1 in promoting CC cell growth under *in vitro* conditions.

### Fascin-1 increases the migration ability of cervical cells *in vitro*

To evaluate the impact of FSCN1 knockdown and overexpression on the migratory capabilities of CC cells, we used HeLa and C33A cells with stable expression of either control or Fascin-1-targeted shRNAs. Transwell assays were performed to assess how reducing Fascin-1 levels affects cell migration and invasion. Our results showed that HeLa and C33A cells with reduced Fascin-1 expression exhibited significantly decreased migration and invasion compared to the control group, as fewer cells traversed the membrane. This suggests that Fascin-1 depletion impairs these cellular behaviors. Figure 3A and 3B clearly illustrate this decline following Fascin-1 suppression. Conversely, overexpression of Fascin-1 in HeLa and C33A cells significantly enhanced their migration and invasion capabilities, as depicted in Figure 3C and 3D. In summary, these findings underscore the critical role of Fascin-1 in promoting CC cell migration and invasion under controlled conditions.

### FSCN1 knockdown suppresses cervical tumor growth and metastasis *in vivo*

**Figure 4.**
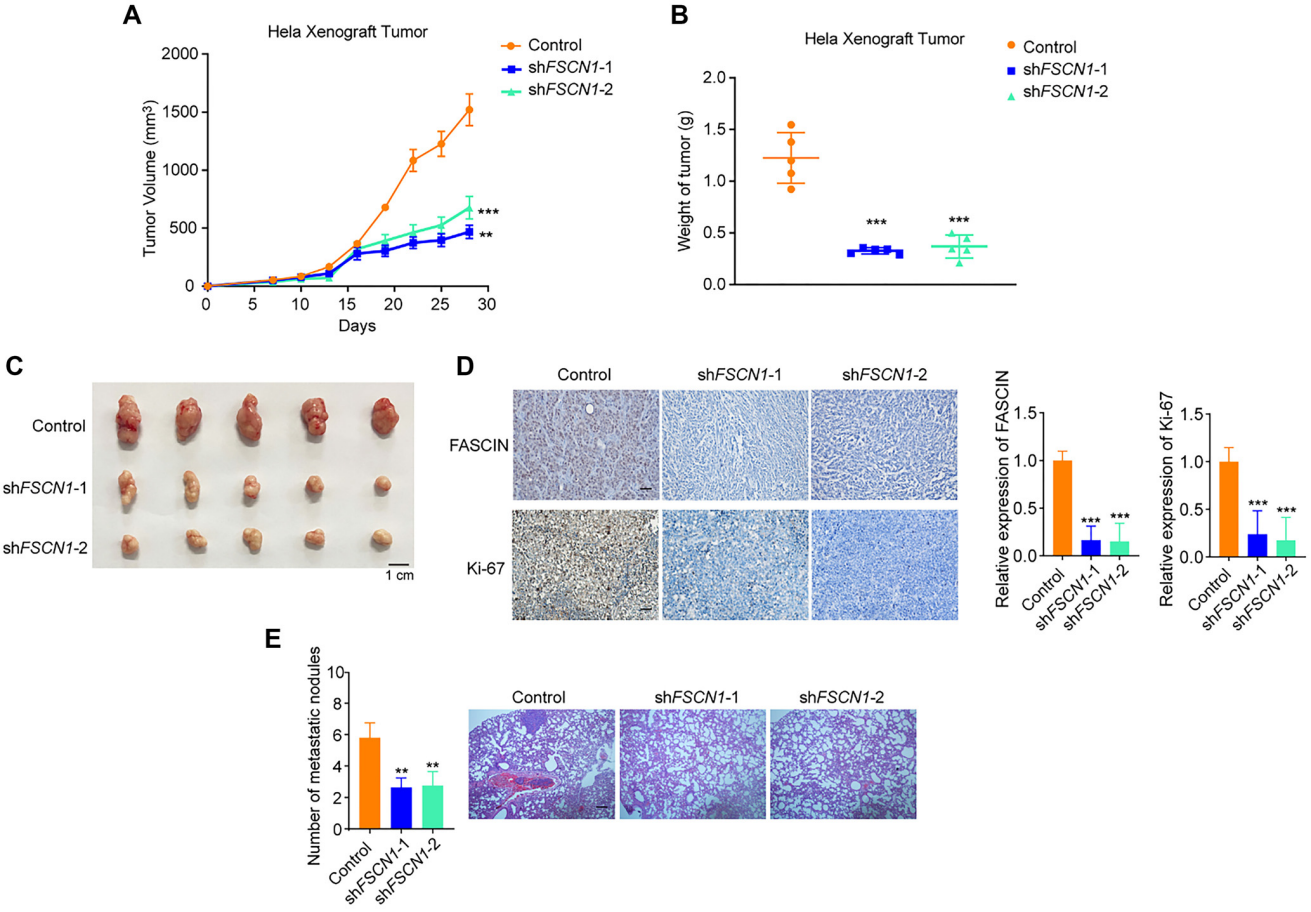
***In vivo* impact of Fascin-1 knockdown on cervical tumor growth and metastasis.** (A) Female athymic nude mice were injected subcutaneously with Hela cells engineered to express either control or *FSCN1*-targeting shRNAs. Tumor dimensions were measured every three days using a vernier caliper, and volumes were computed with the formula: *V* ═ 1/2 × length × width^2^. (B and C) The weight of the tumors was also recorded. For Figure 4A and 4B, each bar represents the average ± SD from five animal measurements; ***P < 0.001, **P < 0.01 compared to control (Student’s *t*-test). (D) Immunohistochemical analysis of frozen tumor sections was conducted using anti-Fascin-1 and anti-Ki67 antibodies. Scale bar is 100 µm. (E) Tail vein injection of BALB/C nude mice with Hela cells expressing control or *FSCN1* shRNAs (*n* ═ 5 per group). The left panel illustrates the statistical analysis of metastatic foci, each bar showing the mean ± SD from five animals; **P < 0.01 versus control (Student’s *t*-test). The right panel shows hematoxylin-eosin (H&E) staining of lung metastases (scale bar: 100 µm).

To further investigate the role of Fascin-1 as an oncogene in CC *in vivo*, we utilized two distinct mouse models: a subcutaneous tumor formation model and a lung metastasis model. These models were selected to assess the impact of Fascin-1 suppression on tumor progression and metastatic spread. In the first phase, nude mice (6–8 weeks old) received subcutaneous injections of either Fascin-1-suppressed HeLa cells or control cells. Tumor progression was systematically monitored every three days. Notably, mice injected with Fascin-1-deficient cells exhibited a significant reduction in both tumor size and mass compared to the control group, as shown in Figure 4A–4C. IHC staining of frozen tumor sections revealed a marked decrease in Fascin-1 and Ki67-positive tumor cells in the Fascin-1 knockdown group (Figure 4D), indicating effective suppression of tumor growth and malignancy. Furthermore, in the lung metastasis model, the number of lung metastases was notably lower in the Fascin-1-silenced group compared to the control group (Figure 4E). In summary, Fascin-1 depletion consistently inhibits CC cell growth and metastasis *in vivo*, aligning with the phenotypic outcomes observed *in vitro*.

### Fascin-1 promotes the Wnt/β-catenin pathway

**Figure 5.**
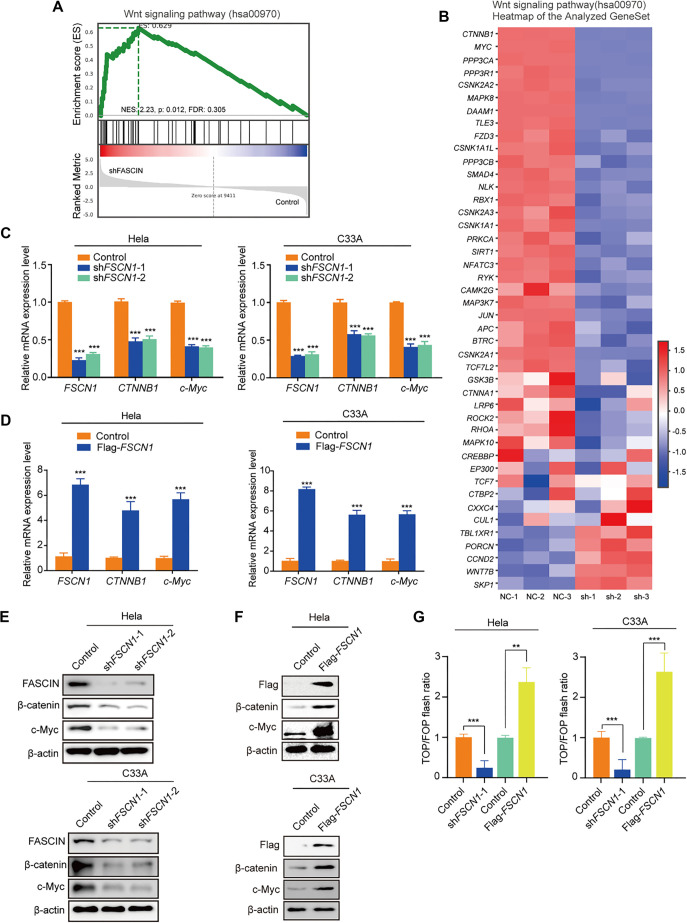
**Downregulation of Fascin-1 impairs Wnt/β-catenin signaling.** (A) GSEA enrichment plot of Wnt signaling pathway using RNA-seq data generated from Hela cells; (B) Heatmap of genes involved in Wnt signaling pathway; (C) mRNA was isolated from Hela and C33A cells expressing specific shRNAs, and quantitative real-time RT-PCR assays were conducted to measure expression levels; (D) RT-PCR analysis of *FSCN1*, *CTNNB1* and *c-Myc* mRNA levels in the indicated cells; (E and F) Protein lysates from indicated Hela and C33A cells, were analyzed by Western blot using relevant antibodies; (G) pTop-Flash/pFop-Flash reporter vectors were used to evaluate the transcriptional activity of the Wnt/β-catenin signaling pathway. Each bar in the graph indicates the average ± SD from three independent experiments, ***P < 0.001 compared to control (Student’s *t*-test). GSEA: Gene set enrichment analysis.

To investigate how Fascin-1 knockdown inhibits CC cell growth and migration, we employed an unbiased genomic approach (RNA-seq) to analyze differential transcriptional programs in fascin-knockdown and wild-type HeLa cells. Gene set enrichment analysis (GSEA) revealed that Fascin-1 knockdown significantly enriched the Wnt pathway in HeLa cell lines (Figure 5A and 5B). Extensive literature has consistently highlighted the crucial role of Wnt/β-catenin signaling in CC pathogenesis, as corroborated by multiple studies [20–23]. Our research aimed to further explore the effects of reduced Fascin-1 expression on CC cell growth and migration. To this end, we analyzed β-catenin and c-Myc—key components of the Wnt/β-catenin pathway—using quantitative real-time RT-PCR (Figure 5C and 5D) and Western blotting (Figure 5E and 5F) in both control and Fascin-1-suppressed CC cell lines. The results showed a marked decrease in β-catenin and c-Myc expression in HeLa and C33A cells following Fascin-1 silencing. Conversely, Fascin-1 overexpression led to increased mRNA and protein levels of β-catenin and c-Myc. Additionally, pTop-Flash/pFop-Flash reporter assays demonstrated that Fascin-1 knockdown inhibited Wnt/β-catenin transcriptional activity, whereas its overexpression enhanced it (Figure 5G). These findings provide deeper insights into the molecular interactions regulated by Fascin-1 and its role in CC progression.

### Knockdown of Fascin-1 sensitizes CC cells to cisplatin

**Figure 6.**
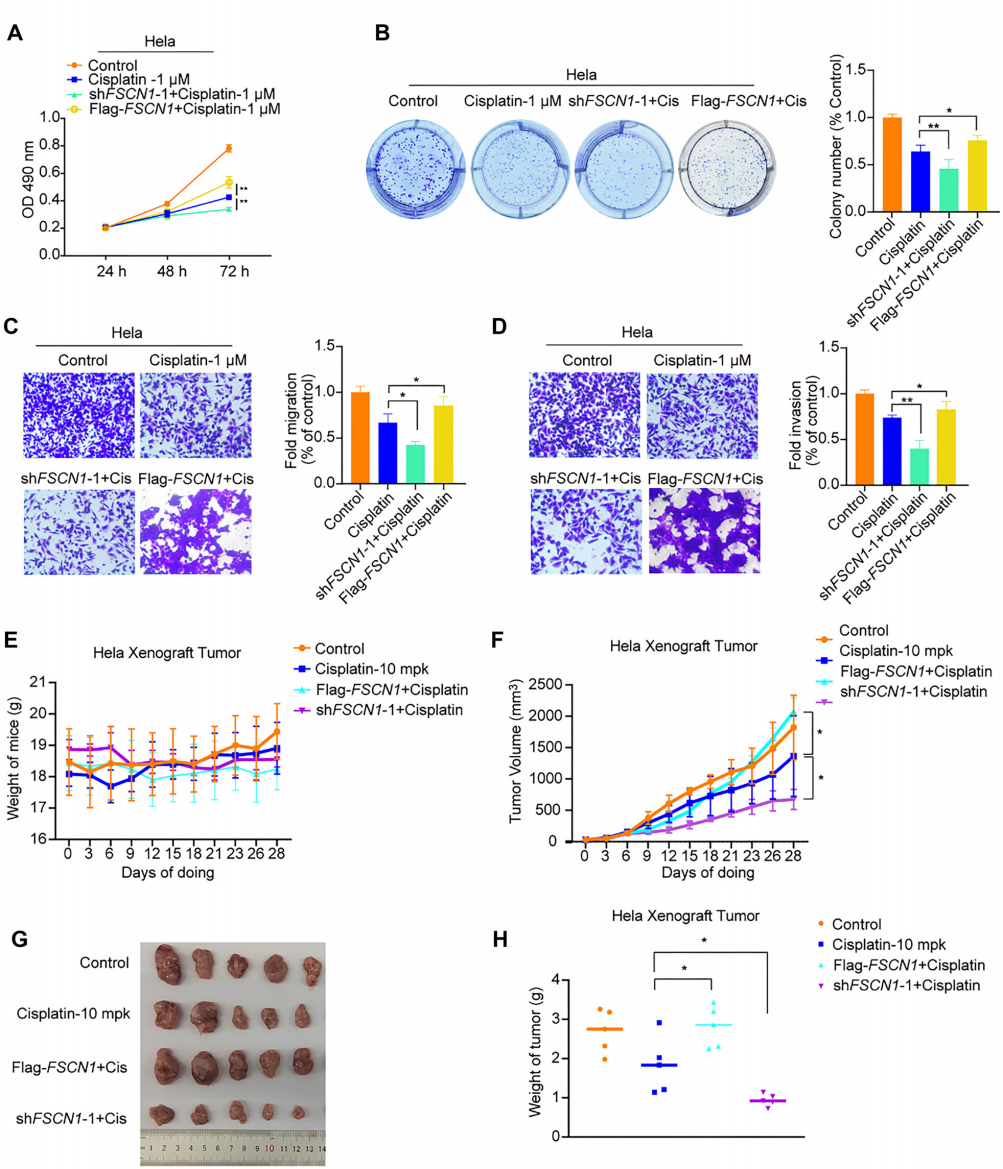
**Enhanced sensitivity to cisplatin in cervical cancer cells via Fascin-1 knockdown.** (A and B) Proliferation and colony formation capacities were assessed using MTT and colony formation assays in the specified cells; (C and D) The cells were also evaluated for their migration and invasion abilities. Scale bars are set at 100 µm; (E) Body weight of test animals; (F) Average tumor volume (mm^3^); (G) Representative image of the tumors; (H) Weight of tumors. Data for each bar represent the mean ± S.D (*n* ═ 5). **P < 0.01, *P < 0.05 compared to control (Student’s *t*-test).

Cisplatin is the most commonly used chemotherapy drug to improve survival rates in CC patients [24–26]. This study aimed to determine whether Fascin-1 knockdown affects the effectiveness of cisplatin treatment in cervical cancer. MTT and colony formation assays revealed that reducing Fascin-1 levels made cervical cancer cells more responsive to cisplatin treatment. However, this increased sensitivity was partially counteracted by the overexpression of Fascin-1 (Figure 6A and 6B). Additionally, migration and invasion assays demonstrated that inhibiting Fascin-1 expression in the presence of cisplatin further suppressed the migration and invasion of HeLa cells, while Fascin-1 overexpression partially reversed this effect (Figure 6C and 6D). To further investigate these findings *in vivo*, we conducted experiments using mice. Specific cells were injected subcutaneously into 6–8-week-old nude mice. Once tumor size reached 80–100 mm^3^, the mice were randomized into treatment groups and administered antitumor drugs. Tumor size was measured every three days. After 28 days, the mice were euthanized, and their tumors were excised, weighed, and photographed. The results showed that the mice’s body weight remained within a reasonable range throughout the experiment (Figure 6E). Additionally, Fascin-1 knockdown significantly enhanced tumor sensitivity to cisplatin, leading to a substantial reduction in tumor volume and weight (Figure 6F–6H).

## Discussion

Fascin-1 plays a pivotal role in gynecological cancers, with its expression levels correlated to the clinical stage and prognosis of cancer patients [27]. In our study, data analysis from the TCGA database revealed that FSCN1 expression was significantly elevated in cervical cancer cases. Moreover, FSCN1 expression showed a strong positive correlation with overall survivalin CC patients, based on TCGA data. We hypothesized that Fascin-1 could serve as a potential biomarker and therapeutic target for CC. Our findings demonstrated that Fascin-1 deletion significantly inhibited the growth and metastasis of cervical cancer cells both *in vitro* and *in vivo*. The Wnt/β-catenin signaling pathway is closely linked to cancer and plays a crucial role in various biological processes, including embryonic development, tissue homeostasis, and cellular proliferation and differentiation under pathological conditions. However, aberrant activation of this pathway is associated with cancer initiation, progression, and malignant transformation [28]. Previous studies have shown that high Fascin-1 expression in breast cancer can activate the Wnt/β-catenin pathway via Focal Adhesion Kinase (FAK), thereby promoting cancer progression [29]. Our results indicate that Fascin-1 knockdown transcriptionally downregulates β-catenin expression, which in turn reduces the expression of c-Myc and suppresses Wnt signaling in CC cell lines. Conversely, Fascin-1 overexpression upregulates β-catenin and c-Myc expression. Furthermore, we demonstrated that Fascin-1 expression in cervical cancer cells is closely associated with sensitivity to cisplatin chemotherapy. Knocking down Fascin-1 expression significantly enhances the efficacy of cisplatin chemotherapy both *in vitro* and *in vivo*. Given its high expression in cervical cancer tissues, Fascin-1 has the potential to serve as a promising biomarker and therapeutic target for anti-cervical cancer treatment. Targeting Fascin-1 through drug development or therapeutic strategies could inhibit cervical cancer cell proliferation and metastasis, thereby improving treatment efficacy and patient survival rates. Although specific therapeutic drugs targeting Fascin-1 are not yet widely used in clinical practice, its potential in cervical cancer treatment has garnered significant attention. The Fascin-1 inhibitor NP-G2-044/DC05F01 is currently undergoing clinical evaluation for solid tumors in phase II trials. With continued research, it is anticipated that targeted therapies against Fascin-1 will be developed, offering cervical cancer patients more treatment options and improved outcomes.

## Conclusion

In conclusion, this study demonstrates that Fascin-1 functions as an oncogene in the malignant progression of cervical cancer. We also uncovered the molecular mechanism by which Fascin-1 promotes the proliferation, migration, and drug resistance of cervical cancer cells by activating the Wnt/β-Catenin signaling pathway. More importantly, Fascin-1 has been identified as a potential biomarker. Our findings suggest that its overexpression may indicate increased resistance of cervical cancer cells to platinum-based chemotherapy drugs, underscoring its clinical significance.

## Supplemental data

Supplemental data is available at the following link: https://www.bjbms.org/ojs/index.php/bjbms/article/view/12114/3780.

## Data Availability

The data generated in the present study may be available from the article.

## References

[1] Podwika SE, Duska LR (2023). Top advances of the year: cervical cancer. Cancer.

[2] Liao S, Deng D, Hu X, Wang W, Li L, Li W (2013). HPV16/18 E5, a promising candidate for cervical cancer vaccines, affects SCPs, cell proliferation and cell cycle, and forms a potential network with E6 and E7. Int J Mol Med.

[3] Siegel RL, Miller KD, Wagle NS, Jemal A (2023). Cancer statistics, 2023. CA Cancer J Clin.

[4] Yadav A, Yadav S, Alam MA (2023). Immunotherapies landscape and associated inhibitors for the treatment of cervical cancer. Med Oncol (Northwood, London, England).

[5] Liu C, Li X, Huang Q, Zhang M, Lei T, Wang F (2023). Single-cell RNA-sequencing reveals radiochemotherapy-induced innate immune activation and MHC-II upregulation in cervical cancer. Signal Transduct Target Ther.

[6] Duenas-Gonzalez A (2023). Combinational therapies for the treatment of advanced cervical cancer. Expert Opin Pharmacother.

[7] van Meir H, Kenter GG, Burggraaf J, Kroep JR, Welters MJP, Melief CJM (2014). The need for improvement of the treatment of advanced and metastatic cervical cancer, the rationale for combined chemo-immunotherapy. Anti-Cancer Agents Med Chem.

[8] Lin S, Taylor MD, Singh PK, Yang S (2021). How does fascin promote cancer metastasis?. FEBS J.

[9] Lin S, Taylor MD, Singh PK, Yang S (2023). Fascin-1 in cancer cell metastasis: old target-new insights. Int J Mol Sci.

[10] Lin S, Li Y, Wang D, Huang C, Marino D, Bollt O (2021). Fascin promotes lung cancer growth and metastasis by enhancing glycolysis and PFKFB3 expression. Cancer Lett.

[11] Li J, Zhang S, Pei M, Wu L, Liu Y, Li H (2018). FSCN1 promotes epithelial-mesenchymal transition through increasing snail1 in ovarian cancer cells. Cell Biochem.

[12] Adams JC (2015). Fascin-1 as a biomarker and prospective therapeutic target in colorectal cancer. Expert Rev Mol Diagn.

[13] Wang CQ, Tang CH, Chang HT, Li XN, Zhao YM, Su CM (2016). Fascin-1 as a novel diagnostic marker of triple-negative breast cancer. Cancer Med.

[14] Zhang H, Xu L, Xiao D, Xie J, Zeng H, Cai W (2006). Fascin is a potential biomarker for early-stage oesophageal squamous cell carcinoma. J Clin Pathol.

[15] Li A, Morton JP, Ma Y, Karim SA, Zhou Y, Faller WJ (2014). Fascin is regulated by slug, promotes progression of pancreatic cancer in mice, and is associated with patient outcomes. Gastroenterology.

[16] Barnawi R, Al-Khaldi S, Majed Sleiman G, Sarkar A, Al-Dhfyan A, Al-Mohanna F (2016). Fascin is critical for the maintenance of breast cancer stem cell pool predominantly via the activation of the notch self-renewal pathway. Stem Cells (Dayton, Ohio).

[17] Ghebeh H, Al-Khaldi S, Olabi S, Al-Dhfyan A, Al-Mohanna F, Barnawi R (2014). Fascin is involved in the chemotherapeutic resistance of breast cancer cells predominantly via the PI3K/Akt pathway. Brit J Cancer.

[18] Ma L, Li LL (2019). miR-145 contributes to the progression of cervical carcinoma by directly regulating FSCN1. Cell Transpl.

[19] Koay MH, Crook M, Stewart CJ (2014). Fascin expression in cervical normal squamous epithelium, cervical intraepithelial neoplasia, and superficially invasive (stage IA1) squamous carcinoma of the cervix. Pathology.

[20] Xu T, Zeng Y, Shi L, Yang Q, Chen Y, Wu G (2020). Targeting NEK2 impairs oncogenesis and radioresistance via inhibiting the Wnt1/β-catenin signaling pathway in cervical cancer. J Exp Clin Cancer Res.

[21] Chi C, Hou W, Zhang Y, Chen J, Shen Z, Chen Y (2023). PDHB-AS suppresses cervical cancer progression and cisplatin resistance via inhibition on Wnt/β-catenin pathway. Cell Death Dis.

[22] Hsu W, Liu L, Chen X, Zhang Y, Zhu W (2019). LncRNA CASC11 promotes the cervical cancer progression by activating Wnt/beta-catenin signaling pathway. Biol Res.

[23] Lei D, Yang WT, Zheng PS (2021). HOXB4 inhibits the proliferation and tumorigenesis of cervical cancer cells by downregulating the activity of Wnt/β-catenin signaling pathway. Cell Death Dis.

[24] Bhattacharjee R, Dey T, Kumar L, Kar S, Sarkar R, Ghorai M (2022). Cellular landscaping of cisplatin resistance in cervical cancer. Biomed Pharmacother.

[25] Federico C, Sun J, Muz B, Alhallak K, Cosper PF, Muhammad N (2021). Localized delivery of cisplatin to cervical cancer improves its therapeutic efficacy and minimizes its side effect profile. Int J Radiat Oncol Biol Phys.

[26] Hung JY, Yang CJ, Tsai YM, Huang HW, Huang MS (2008). Antiproliferative activity of aucubin is through cell cycle arrest and apoptosis in human non-small cell lung cancer A549 cells. Clin Exp Pharmacol Physiol.

[27] Gupta I, Vranic S, Al-Thawadi H, Al Moustafa AE (2021). Fascin in gynecological cancers: an update of the literature. Cancers.

[28] Xue W, Yang L, Chen C, Ashrafizadeh M, Tian Y, Sun R (2024). Wnt/β-catenin-driven EMT regulation in human cancers. Cell Mol Life Sci.

[29] Barnawi R, Al-Khaldi S, Bakheet T, Fallatah M, Alaiya A, Ghebeh H (2020). Fascin activates β-catenin signaling and promotes breast cancer stem cell function mainly through focal adhesion kinase (FAK): relation with disease progression. Front Oncol.

